# The application of mechanobiotechnology for immuno-engineering and cancer immunotherapy

**DOI:** 10.3389/fcell.2022.1064484

**Published:** 2022-11-22

**Authors:** Chi Woo Yoon, Yijia Pan, Yingxiao Wang

**Affiliations:** Department of Bioengineering, Institute of Engineering in Medicine, University of California, San Diego, San Diego, CA, United States

**Keywords:** mechanobiology, mechanotransduction, immune cells, immuno-engineering, focused ultrasound

## Abstract

Immune-engineering is a rapidly emerging field in the past few years, as immunotherapy evolved from a paradigm-shifting therapeutic approach for cancer treatment to promising immuno-oncology models in clinical trials and commercial products. Linking the field of biomedical engineering with immunology, immuno-engineering applies engineering principles and utilizes synthetic biology tools to study and control the immune system for diseases treatments and interventions. Over the past decades, there has been a deeper understanding that mechanical forces play crucial roles in regulating immune cells at different stages from antigen recognition to actual killing, which suggests potential opportunities to design and tailor mechanobiology tools to novel immunotherapy. In this review, we first provide a brief introduction to recent technological and scientific advances in mechanobiology for immune cells. Different strategies for immuno-engineering are then discussed and evaluated. Furthermore, we describe the opportunities and challenges of applying mechanobiology and related technologies to study and engineer immune cells and ultimately modulate their function for immunotherapy. In summary, the synergetic integration of cutting-edge mechanical biology techniques into immune-engineering strategies can provide a powerful platform and allow new directions for the field of immunotherapy.

## Introduction

Cell mechanobiology is a rapidly developing field at the interface of biology, physics, and engineering (Ranade et al., 2015; Chien et al., 2016). It studies how physical cues and mechanical properties of cells contribute to initiating and maintaining cellular functions and inducing pathophysiological changes in cells. Although all types of cells in the body are virtually exposed to mechanical environments, most of the current mechanobiology research has been focused on stem cells, cancer cells, and cells in the cardiovascular system and the skeletal muscular system. For example, shear stress regulates vascular tones in health and disease status through vascular endothelial cells (ECs) mechanotransduction (Li et al., 2005). Stem cells are regulated by mechanical cues from their surrounding microenvironment for development, homeostasis, and differentiation (Kim et al., 2015; Shin and Mooney, 2016). Changes in the extracellular matrix (ECM) mechanics, cytoskeleton tension, and mechanotransduction signaling also are important factors for tumorigenesis and malignant transformation (Chin et al., 2016; Northcott et al., 2018). The importance of studying mechanobiology is not only for understanding the role of mechanical forces in cellular regulation but also for discovering potential targets for diseases and designing novel therapeutic tools. Although recent studies have begun to shed light on the underlying mechanism of mechanobiology in immune cells (Zhu et al., 2019), the roles of mechanical force in regulating immune cells, despite its importance, remain relatively less explored.

Immuno-engineering aims to apply engineering strategies to advance the discovery and therapeutic manipulation of the immune system (Swartz et al., 2012). One major goal of immuno-engineering is to develop new molecular and cellular immunotherapies. In addition, immuno-engineering also contributes to our fundamental understanding of immunology. The advantage of immune-engineering is to bring more synthetic, quantitative, and translationally oriented analysis and solutions into the complicated immune system, therefore allowing us to harness the powerful tumor-fighting capacity of the immune system with more precise control.

Here, we summarize the recent progress in understanding the mechano-sensing and mechano-regulation in immune cells, review current immunotherapy strategies, discuss the applications of mechanobiology in immunotherapy, and project how these insights may be used to translate to future immunotherapy applications.

## Mechanobiology in immune cells

Immune cells help the body fight infections and other diseases. These immune cells include neutrophils, eosinophils, basophils, mast cells, monocytes, macrophages, dendritic cells, natural killer (NK) cells, and lymphocytes (B cells and T cells). In this paper, we will mainly focus on three major immune cell types, namely T cells, NK cells, and macrophages, that have been extensively studied and engineered in cancer immunoengineering field. To learn more about mechanobiology in B cells (Shaheen et al., 2019), or neutrophils (Ekpenyong et al., 2015), please refer to the cited papers. Furthermore, some of major mechanosensing cellular components will be discussed in this chapter.

### Mechanotransduction in T cells

**FIGURE 1:**
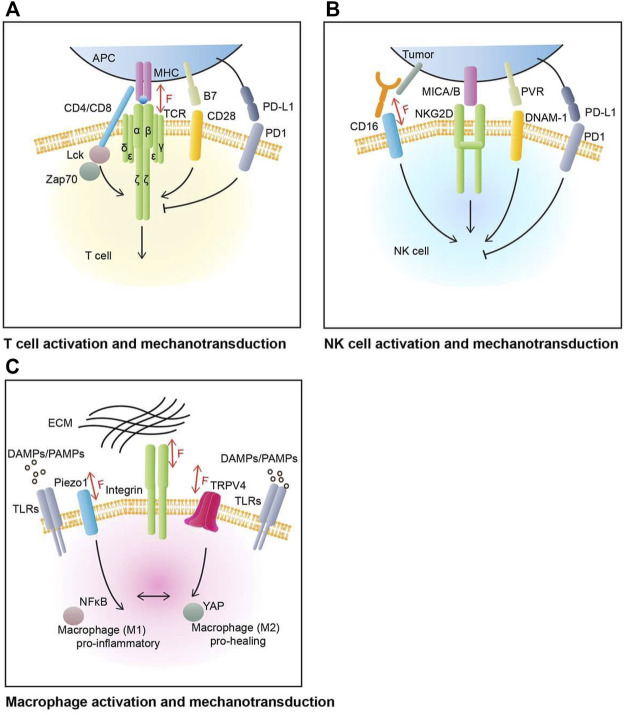
T cell, NK cell, and macrophage activation and mechanotransduction. **(A)** T cell’s main activation and inhibition molecular pathways. The main activation receptor for T cell is TCR, which binds to MHC complex in antigen presenting cells (APCs). CD28 is one of T cell’s co-stimulatory receptor, with its ligand B7. PD1 is one of T cell’s co-inhibitory receptors, with its ligand PD-L1. TCR can sense, mediate, and generate mechanical force through TCR-pMHC complexes. **(B)** NK cell’s major activation and co-stimulation molecular pathways. The activation of NK cell is mediated by the balance of activating and inhibitory receptors network. NK cell main activation molecules include NKG2D (natural killer group 2D) and DNAM-1 (DNAX accessory molecule 1) and some of their ligands PVR and MICA/B (MHC class I polypeptide-related sequence A/B). PD1 is an example of NK cell inhibitory receptor, with its ligand PD-L1 on tumor cells. The CD16 Fc receptors can recognize cell-bound antibodies and trigger tumor cell apoptosis. CD16 can be involved in NK cell mechanosensing. **(C)** Macrophage’s polarization and mechanotransduction. Macrophage respond to various environment cues to perform its housekeeping role. Pathogen-associated molecular patterns (PAMPs) and damage-associated molecular patterns (DAMPs) are major family of signals that induce macrophage’s activation through Toll-like receptors (TLRs). Mechanosensitive ion channels and adhesion proteins on macrophage’s plasma membrane help transducing mechanical input such as topological patterns or stiffness of extracellular matrix (ECM). These inputs will regulate the polarization of macrophage between pro-inflammatory (M1) and pro-healing (M2) phenotypes as well as enhancing phagocytic function of macrophage.

T cells, especially CD8^+^ T cells, as an essential part of the immune system, can detect and kill tumors (Sallusto et al., 2004). T cell activation involves complex molecular interactions, including major T cell activation pathways and co-signaling pathways (Figure 1A). In general, T cell activation is initiated by the recognition and interaction of T cell receptor (TCR)/CD3 complex to peptide-major histocompatibility complex (pMHC) molecules on the antigen-presenting cells (APCs). This activation pathway is typically mediated by Lck and Zap70 and their related signaling molecules (Chen and Flies, 2013). Full T cell activation requires co-stimulatory signaling, mediated by CD28 and other receptors, typically involving PI3K and Akt pathways (Chen and Flies, 2013).

There is increasing evidence and appreciation that mechanical forces can modulate T cell migration, recognition, activation, and subsequent effector functions. T cells are circulating through the body to recognize and target APCs, where they will enter different microenvironments with varied mechanical stiffness and distinct mechanical properties (Rushdi et al., 2020). T cell itself has been reported to be a self-referential mechano-sensor that responds to various mechanical forces (Kim et al., 2009; Judokusumo et al., 2012). For instance, mechanical tension applied to TCR/pMHC interactions prolongs its bond lifetime where both optimal magnitude and duration of force are required for efficient T cell activation (Liu et al., 2014). A recent study indicates that T cells in 3D microporous hydrogels can sense their mechanical environment and amplify effector response under the influence of mechanical stiffness (Majedi et al., 2020). It was found that T cell migration, proliferation, activation, as well as the production of cytokines were significantly higher in a stiff 3D environment. Beyond T cell activation, force exertion at the immunological synapse (IS) potentiates the cytotoxicity of target cells by enhancing perforin activity (Basu et al., 2016).

Current approaches to study T cell mechanotransduction mainly include biophysical tools and engineered extracellular substrates. A series of studies utilized biophysical techniques to exert localized force on T cells to probe mechanosensing, including optical tweezer (Feng et al., 2017), AFM (Hu and Butte, 2016), micropipette (Basu et al., 2016), and fluorescence biomembrane force probe (fBFP) (de la Zerda et al., 2018). The application of localized force by these strategies has the advantage of precise spatial and temporal control to quantitatively study immune cell mechanotransduction at the single-cell level. One limitation of these techniques with high spatiotemporal perfection is that such stimulation can only be applied for a short period of time. On the other side, engineered substrates including 2D and 3D scaffolds composed of different types of hydrogels (Saitakis et al., 2017), micropillars (Basu et al., 2016), or micro-/nano-scale patterned substrates with varied rigidities (Jin et al., 2019) have been applied to evaluate T cell responses to mechanical environment changes over a longer time period (de la Zerda et al., 2018; Jin et al., 2019). However, 2D and 3D scaffolds have limitations, e.g., the exerted force cannot be directly measured or precisely controlled.

### Mechanotransduction in natural killer cells

CD8^+^ T cells and natural killer cells are both cytotoxic effector cells in the immune system. While extensive studies have been focusing on CD8^+^ T cells, little is known about NK cells. NK cells are a type of cytotoxic lymphocytes that contain features of both innate and adaptive immunity (Netea et al., 2016) and they play important roles in innate immune responses. Unlike T cells that utilize a highly specific TCR to identify distinct antigens, NK cells do not possess a single dominant receptor to mediate recognition. Instead, NK cells express an array of innate activating or inhibiting receptors to sense their environment and respond with activation or inhibition (Huntington et al., 2020) (Figure 1B). While a few models have been proposed (Kim et al., 2005), the molecular mechanism of how NK cells determine their recognition and activation remains unclear. Two known major NK cell activation mechanisms are activation receptor NKG2D mediated activation, and CD16 mediated antibody-dependent cellular cytotoxicity (ADCC) (Paul and Lal, 2017).

Compared to T cells, NK cell mechanotransduction is much less studied. A few recent studies have explored the mechanosensing of NK cells. For example, a recent study suggests that actomyosin retrograde flow (ARF) controls NK cell immune response through the interaction between *β*-Actin and the SH2 domain-containing protein kinase phosphatase 1 (SHP-1) (Matalon et al., 2018). Another study utilized two anti-CD16 nanobodies (C21 and C28) targeting different epitopes of CD16 on NK cells in a laminar flow chamber assay and C21-CD16 pair demonstrated a slip bond behavior while the other showed a catch-bond dissociation pattern, providing evidence of mechanosensing in NK cells (Gonzalez et al., 2019).

### Mechanotransduction in macrophages

A macrophage is a type of white blood cell that plays a critical role in innate immunity. Known as “big eaters,” macrophages take phagocytic actions against pathogens, dying cells, and debris. However, recent discoveries revealed their roles in a vast range of cellular processes, including tissue hemostasis, development, and repair (Okabe and Medzhitov, 2016). Accordingly, macrophage dysfunction is often associated with the progression of various inflammatory diseases (Jain et al., 2019). To perform their housekeeping duty, instead of working alone, macrophages interact with other types of cells as well as the ECM through active sensing and processing of information (Mosser et al., 2021). In response to various chemical and physical inputs, macrophages flexibly alter their polarization between M1 (pro-inflammatory) and M2 (pro-healing) phenotypes to adapt and function accordingly (Orecchioni et al., 2019) (Figure 1C).

There has been cumulative evidence suggesting the mechanical and physical regulation of macrophage polarization and function. An early study demonstrated that, by showing enhanced phagocytosis through the formation of F-actin, macrophages react to topographic features of a nanometric scale, which is comparable to a single collagen fiber (Wojciak-Stothard et al., 1996). The enhanced phagocytosis is also observed in the macrophages cultured on the stiff substrate, *via* an altered cell elasticity (Patel et al., 2012). In addition, cell elasticity, modulated by substrate rigidity, was also found to regulate macrophage transcriptome profiles as well as its inflammatory responses. A recent study discovered that a well-established player in mechanotransduction, yes-associated protein (YAP), is responsible for mediating the inflammatory activation of macrophages in response to substrate stiffness (Meli et al., 2020). Beyond stiffness sensing, cell morphology and spatial confinement are also found to be major factors for macrophage plasticity (McWhorter et al., 2013; Jain and Vogel, 2018).

### Mechanosensitive ion channels in immune cells

Mechanically-gated ion channels are often considered primary cellular mechano-sensors allowing the exchange of several cations in and out of the cells upon channel activation *via* membrane tension (Martinac, 2004). In particular, calcium channels play pivotal roles in mechanotransduction as nearly every cellular event is associated with intracellular calcium signaling (Clapham, 2007), and immune cells are no exception (Vig and Kinet, 2009). Among the ion channels that are found to be sensitive to mechanical cues, Piezo1 has gained significant attention since its first identification as a mechano-sensor in 2010 (Coste et al., 2010). In humans, Piezo1 is widely distributed across the body and plays an important role in mechano-regulation, particularly in cardiovascular, lung, and urinary (Wu et al., 2017).

**FIGURE 2:**
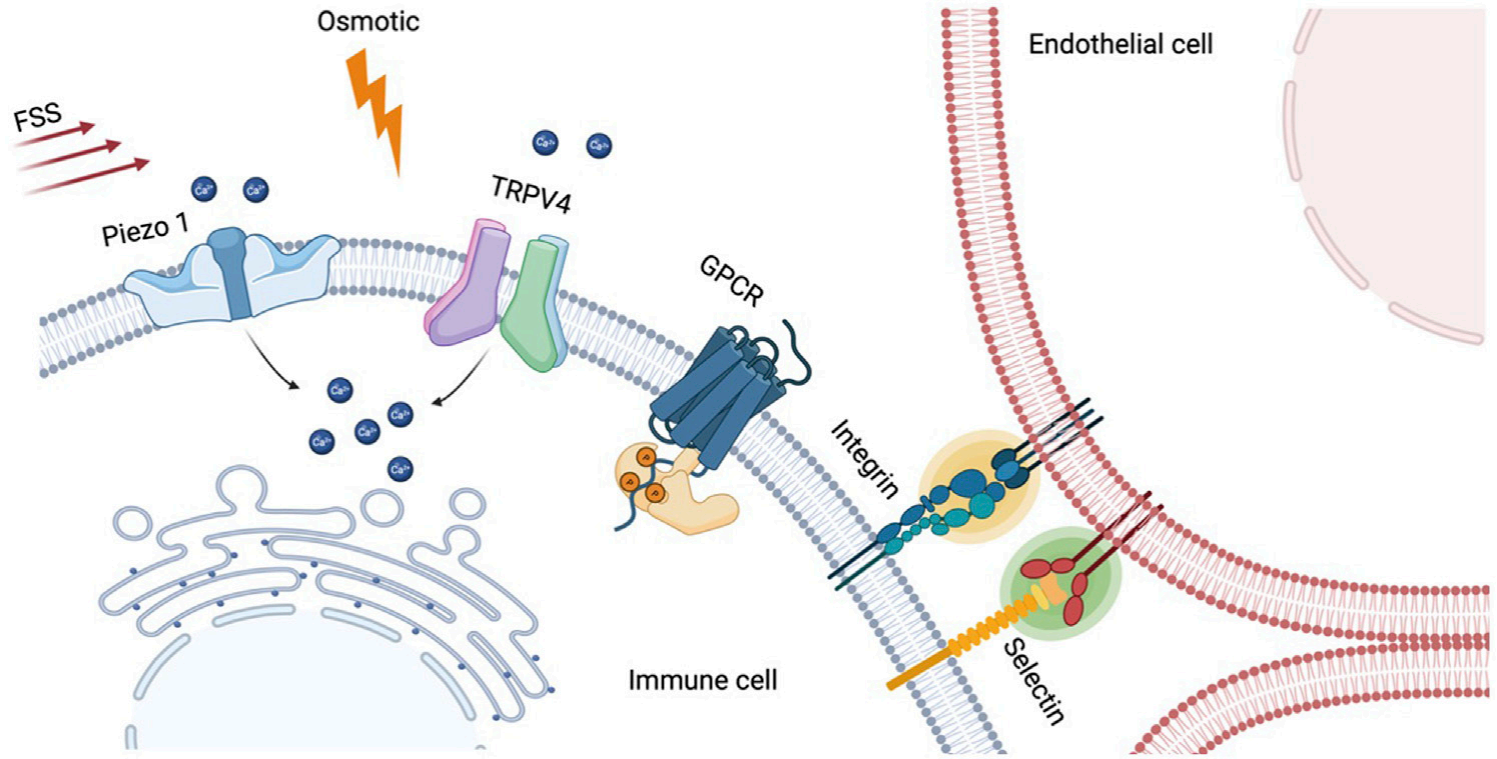
Various mechanosensitive cellular components in immune cells. Mechanically-gated ion channels such as Piezo1 and TRPV4 allow ion influx (e.g., Ca^2+^) in response to membrane tension generated by fluid shear stress (FSS), osmotic shock, or ligand binding through adhesion molecules. The resulting calcium elevation was found to orchestrate a broad range of immune cell functions including activation, differentiation, and polarization. Non-channel type mechanosensitive components also play role in immune cells. Cell adhesion proteins, selectin and integrin, are the well-established mechanosensors, and their bindings to endothelial cells in T cell rolling and arrest can be enhanced by laminar blood flow. Lastly, G protein-coupled receptors (GPCRs), a group of cellular sensors to diverse extracellular stimuli, can also be biosensors for mechanotransduction pathway in circulating immune cells.

There have been several reports illustrating the major contribution of Piezo1 to immune function particularly in T cell activation and polarization (Figure 2). For instance, Liu et al. (2018) reported that mechanical tension at TCR/pMHC interaction can be transmitted *via* Piezo1-mediated calcium responses for optimal T cell activation. Significant reduction of early T cell activation markers, phosphorylation of ZAP70 and CD69 expression, were observed in Piezo1-deficient T cells. Meanwhile, supplementation with Piezo1 agonist bypasses the need for mechanical tension to obtain optimal T cell activation. Similar lines of evidence were also reported recently that fluid shear stress (FSS) can enhance the T cell activation *via* Piezo1 mediated calcium signaling reassuring the importance of the mechano-sensor in T cell function (Hope et al., 2022). Beyond T cell activation, Piezo1 was also found to be mediating helper T cell polarization. A recent paper demonstrated that deletion of Piezo1 in CD4^+^ T cells leads to expansion of the regulatory T (Treg) cell population through enhanced TGF-β signaling (Jairaman et al., 2021).

Piezo1 plays important roles in innate immune cells as well. Solis et al. (2019) showed that cyclic hydrostatic pressure alone, which mimics the dynamic environment of the lung, can activate proinflammatory responses in myeloid cells through Piezo1-mediated calcium influx. Impaired host defense against bacterial infection was observed in mice with Piezo1-deleted myeloid cells, highlighting the physiological importance of Piezo1-dependent mechanosensation in innate immunity. Immunoregulatory role of Piezo1 signaling in myeloid cells was also explored in cancer. The progression level of pancreatic ductal adenocarcinoma (PDA) is closely correlated to the global Piezo1 activity, and Piezo1 in myeloid cells promotes cancer progression (Aykut et al., 2020). These findings suggest that Piezo1 can be a promising target for PDA treatment. Lastly, in macrophages, mechanotransduction *via* Piezo1 signaling is found to be a key regulator for polarization in response to stiff environment (Atcha et al., 2021).

Another mechanosensitive ion channel worth to note in this context is transient receptor potential vanilloid-type 4 (TRPV4). It was first identified as a non-selective cation channel that respond to osmolarity changes (Liedtke et al., 2000; Strotmann et al., 2000), and later also found to be universal sensor that respond to various stimuli including heat, low pH, and mechanical cues (O’Neil and Heller, 2005; Hamanaka et al., 2007). TPRV4 is ubiquitously expressed in various cells including immune cells such as macrophages and neutrophils and serves as important mediator for their activation and differentiation (Partida-Sanchez et al., 2021). More comprehensive reviews on this mechanosensitive channel can be found in these two recent review papers (Michalick and Kuebler, 2020; Orsini et al., 2021).

### Non-channel type mechanosensors in immune cells

Beside the ion channels, there are several other cellular components that interact with biophysical cues and mediate cellular functions. For instance, cell adhesion molecules, selectins and integrins, plays critical roles as mechanosensing components in T cell rolling and arrest on the endothelium which can be considered as the very first step for T cell localization (Rossy et al., 2018). It was found that shear stress generated by blood flow enhances the adhesion process of T cells on the endothelium, first by selectins for initial capture, followed by integrins for the firm arrest through catch-bond formation (Huse, 2017). Beyond adhesion, integrin also plays role in T cells activation. Lymphocyte function-associated antigen-1 (LFA-1) is a leukocyte-specific integrin on T cells that binds to intracellular adhesion receptor 1 (ICAM-1) on APCs in which their binding amplifies the effect of TCR-clustering in the immunological synapse (Benard et al., 2018; Walling and Kim, 2018). Interestingly, the binding affinity between LFA-1 and ICAM-1 changes as the tension at the binding site increases, indicating mechanosensitive aspect of the integrin (Chen et al., 2010).

G protein-coupled receptors (GPCRs) are the largest family of membrane receptors respond to broad spectrum of extracellular signals (Rosenbaum et al., 2009), and recent accumulating evidences have shed light on mechanosensitive role of the receptors (Lin et al., 2022). In 2006, Makino and her colleagues showed that GPCRs in neutrophils serve as mechanosensor in response to fluid shear stress using FRET imaging (Makino et al., 2006). Since then, several GPCRs are found to be responsible for mechano-sensing in circulating leukocytes such as EMR2 (Boyden et al., 2016), CD97 (Karpus et al., 2013), and GPR56 (Yeung et al., 2020).

## Immuno-engineering strategies

### Chimeric antigen receptor engineering

**FIGURE 3:**
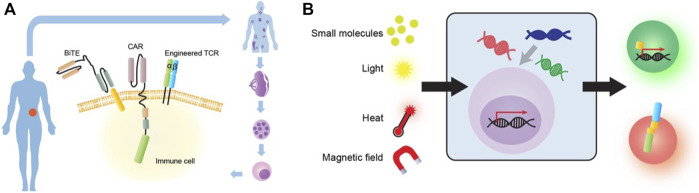
Common immuno-engineering strategies. **(A)** Mainstream immune-engineering strategies at cellular and molecular levels. Immune cells from patients can be engineered with different effector modalities including CAR, TCR and Bispecific T cell engager (BiTE). **(B)** Novel biophysical or synthetic biology-based strategies to engineer immune cells for cancer therapy. The engineered immune cell can be stimulated by various input sources such as small molecules, light, heat and magnetic field. These cells can then process the signal through engineered genetic circuits by transcription factor (TF) (green cell) or dimerization (red cell) to produce functional outputs.

The most well-known immuno-engineering strategy—Chimeric antigen receptor (CAR) expressing T cells, is becoming a paradigm-shifting therapeutic approach for cancer treatment. CAR is a synthetic membrane protein, which consists of a specific antigen targeting extracellular domain, a transmembrane domain, and an intracellular signaling domain for immune cell activation (Jackson et al., 2016) (Figure 3A). The major advantage of CAR is to enable T cells to recognize tumor cells with one single antigen and initiate killing without requiring a complex recognition system. Several generations of CAR have been developed to improve the efficacy and proliferation of the engineered T cells (Guedan et al., 2019). Following successful clinical trials with second-generation autologous CD19-targeted CAR-T cells, FDA approved two products in 2017, Kymriah and Yescarta, to be on the market. Since then, four more products, Abecma, Breyanzi, Carvykti, and Tecartus, has been approved by FDA. Recent analysis also suggests that 96.4% of ongoing clinical trials on CAR products use T cells (MacKay et al., 2020).

The current limitation of autologous CAR-T products includes severe toxicities, patient-dependent variation of T cell qualities, manufacturing time, and high costs (Wang and Riviere, 2016). In contrast, “off-the-shelf” allogeneic CAR-T approach has potential to address the challenges in the autologous CAR products, however it may cause life-threatening graft-versus-host disease (GvHD) and could be eliminated by the host immune systems (Depil et al., 2020). While being at its infancy stage, engineering NK cells with CARs starts to show some promise (Guillerey et al., 2016). Clinical trials with allogenic haploidentical NK cells have been proven to be safe (Liu et al., 2020). NK cells also retain innate anti-tumor cytotoxicity (Huntington et al., 2020). As such, by combining with CARs for targeted cytotoxicity, NK cells can be a promising cell type for CAR designs (Rosenberg and Huang, 2018). Recently, induced pluripotent stem cells (iPSC)-derived T or NK cells have also provided an ideal source for allogenic CAR-T or CAR-NK cell products, due to their versatile engineering potential and unlimited proliferating potentials (Zhu et al., 2020). Recent progress in 3D-organoid based iPSC differentiation platforms may also help facilitate the clinical adaptation of allogeneic CAR approaches (Montel-Hagen et al., 2019; Wang et al., 2022).

Despite its remarkable success in treating hematological malignancies, CAR T cell therapy was not as successful in treating solid tumors (Ma et al., 2019). The notorious immunosuppressive tumor microenvironment (TME), along with other obstacles, greatly reduces the efficacy of CAR T against solid tumors. To circumvent this problem, researchers turn their attention to macrophages as CAR carriers due to their abundant presence in the TME. Indeed, engineering macrophages with CAR to redirect their phagocytic action toward antigen-presenting tumor cells has shown superior tumor control *in vitro* and *in vivo* (Klichinsky et al., 2020). Besides its phagocytic action, it was found that CAR macrophages (CAR-Ms) also induce M1 conversion of bystander M2 macrophages. Furthermore, CAR-Ms can also activate T cells by serving as APCs, essentially turning a “cold tumor” into a “hot tumor” with signs of inflammation. Currently, a phase 1 clinical trial is ongoing for CAR-M therapy against HER2-positive solid tumors. In addition, iPSC derived macrophages (iPSC-Macs) that express a CAR have also been demonstrated to effectively kill tumor cells *in vitro* and *in vivo*, which provides potential methods for developing off-the-shelf cell therapy products (Pouyanfard et al., 2021).

### TCR, BiTE, and other small molecules based on synthetic biology

Another major target effector module for immuno-engineering is TCR (Figure 3A). In comparison to CAR, TCR can recognize intracellular antigen fragments presented by MHC molecules. TCR-engineered T cell (TCR-T) immunotherapies are currently undergoing phase 1 and 2 clinical trials (Zhang and Wang, 2019). There are two main advantages of TCR-T immunotherapy. First, unlike CAR, TCR can recognize a broad range of targets, including both antigens expressed on the cell surface and intracellularly (Zhao and Cao, 2019). Second, TCR can efficiently detect and amplify antigenic signals even at a low copy number (Garber, 2018). Moreover, a recent study identified a new TCR that specifically kills many types of cancer cells *via* recognition of the non-polymorphic MHC class I-related protein (MR1). MR1 is widely expressed in different cancer cells and varies little between individuals, which offers opportunities for pan-cancer immunotherapies that can simultaneously target diverse tumor types (Crowther et al., 2020). In addition, TCR and CAR-Single-chain variable fragment (ScFv) fusion constructs have also been explored by another study for anti-tumor activity (Baeuerle et al., 2019). While having its unique advantages, TCR-T has some inherent limitations including TCR mispairing, MHC restriction, and cytokine toxicities (Zhao and Cao, 2019).

Bispecific T cells engager (BiTE) is another promising engineering strategy for immunotherapy (Figure 2A). A BiTE is a recombinant bispecific protein that has two linked ScFvs from two different antibodies, one targeting a surface molecule on T cells, and the other targeting an antigen on tumor cells (Goebeler and Bargou, 2020). CD3 is the most used targeting molecule on T cells. The power of the BiTE strategy is that it enables the engagement of cytotoxic T cells with cancer cells through BiTE connection to induce cancer cell-specific lysis, independent of T cell receptor specificity, co-stimulation, or peptide antigen presentation. Thus, one major advantage for BiTE molecules is their potential for off-the-shelf products because they no longer rely on engineering specific T cell clones (Baeuerle and Reinhardt, 2009). Nonetheless, BiTEs also face several challenges including short serum half-life, patient-dependent variation of T cell qualities, and toxicities.

Other than engineered effector modules, small molecules mediated synthetic biology has been used for more controlled immuno-engineering (Figure 3B). Small hetero-dimerizers such as rapalog and gibberellin have been used to bring together the engineered split CARs, exclusively in the presence of these dimerizers, to achieve temporally and spatially controllable CAR function (Wu et al., 2015). A similar strategy using a leucine zipper has also been tested for split CARs (Cho et al., 2018). In addition, FDA-approved small molecules 4-hydroxy-tamoxifen (4-OHT) and 33-MB-PP1 have been applied to respectively activate or inhibit a synthetic ZAP70 (Wong and Wong, 2018) to control TCR signaling.

### Light- and temperature-based immunoengineering

Besides traditional biomolecular and biochemical approaches, other novel biophysical engineering strategies have been integrated into immuno-engineering (Figure 3B). The integration of light-sensitive domains in protein engineering has given rise to the field of optogenetics, which revolutionized genetic engineering and therapeutic applications (Deisseroth, 2015). These light-sensitive domains vary in their wavelength sensitivity, photochemical kinetics, and activation mechanism, therefore providing a versatile tool for immune-engineering. Recently we developed an inducible CAR T therapy which uses blue light to control nuclear translocation and dimerization of a synthetic transactivator which activate CAR expression in primary T cells and consequently control tumor growth in mice (Huang et al., 2020). The concept of light-inducible CAR-T therapy provides unprecedent precision to the cell-based therapy where its activation could be tightly controlled by the external modulator. However, clinical adaptation of optogenetic approaches in general is still largely limited by light’s short penetration depth.

Similar to light, the integration of temperature-sensitive domains in protein engineering has also been adopted to engineer heat-inducible expression system. For instance, Yamaguchi et al. (2014) used magnetic nanoparticles which generate heat in response to an alternating magnetic field to drive heat-sensitive protein (HSP) promoter system. More recently, several other groups developed HSP-based immunotherapies by using plasmonic gold nanorods coupling to near-infrared light (Miller et al., 2021), or focused ultrasound (Wu et al., 2021; Abedi et al., 2022) to generate localized heat. It is worth to note that, among the modalities listed above, focused ultrasound does not require additional cofactor injected into the body to increase temperature in the targeted region. The benefit of using ultrasound as external modulator in cell-based therapy will be discussed in the next chapter in details. However, HSP-based genetic circuit is also responsive to variety of stresses, and heat could also induce non-specific thermos-bioeffects. Nevertheless, these novel strategies have shown promise in advancing remotely and precisely controllable immunotherapies.

## Apply mechanobiology to engineer immune cells

Recent evidence and deepened understanding of how cells interact with physical environments have paved the way for the development of mechano-based therapeutic approaches. Synthetic biology and genetic engineering toolkits were utilized to engineer cells to respond to different physical cues by introducing synthetic receptors and/or rewiring endogenous signaling cascades (Hughes and Kumar, 2016). In this approach, mechanical inputs can be originated not only internally by native cellular environments but also externally by mechanical energy-based modalities, which adds an additional level of controllability. Mechano-based therapeutic designs can also be incorporated with previously developed chemical- or light-based systems to provide higher specificity which is an essential requirement for safer cell therapy.

Given the fact that immune cells have mechanosensing and mechanotransduction capabilities, mechanobiology concepts and understandings can be useful tools to engineer immune cells for fundamental research or therapeutic purposes. Here we review techniques to apply mechanobiology to engineer immune cells for therapeutic purposes.

### Synthetic mechanosensing and molecular modulators

**FIGURE 4:**
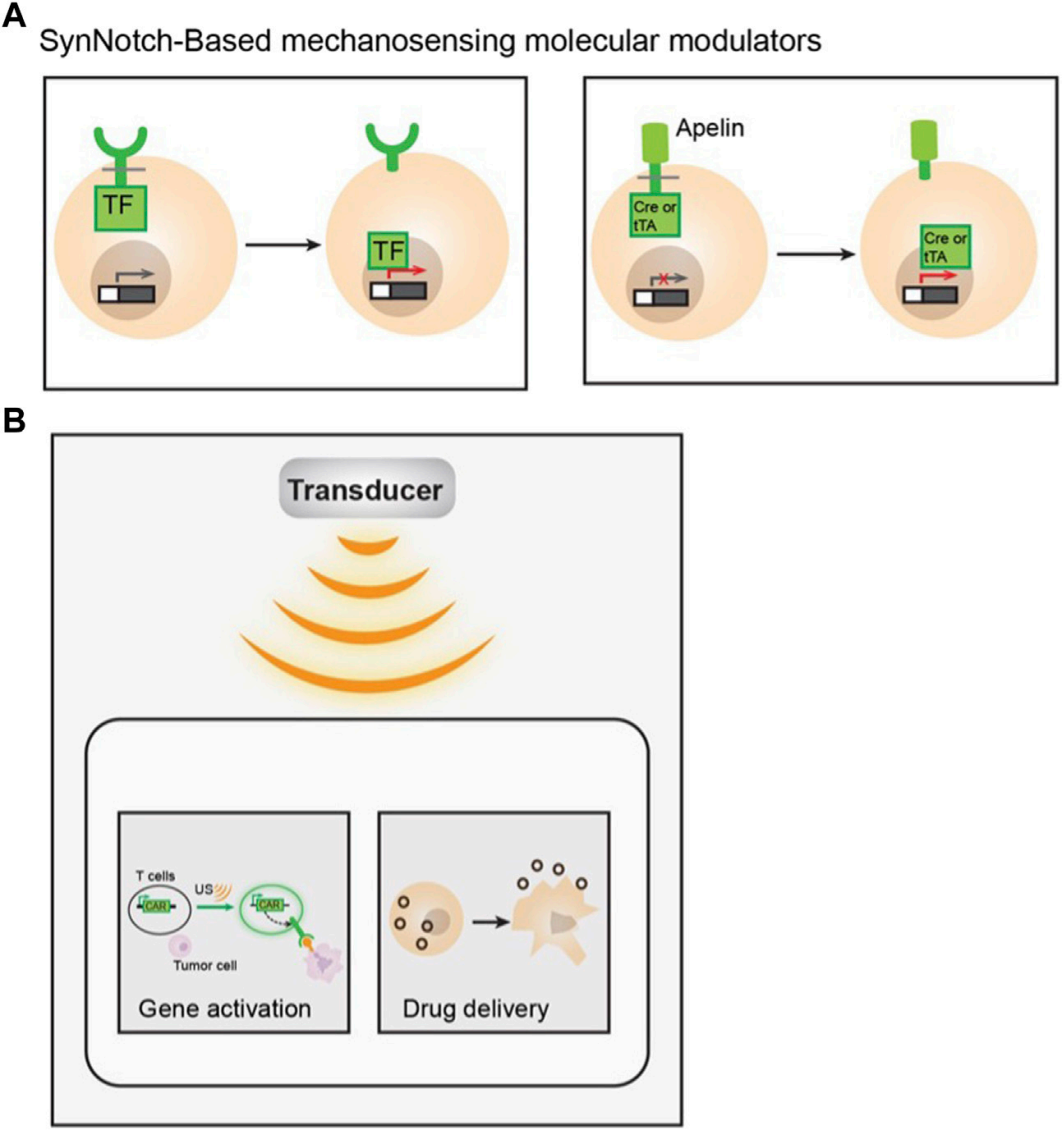
Examples of applying mechanobiology for immuno-engineering. **(A)** Examples of SynNotch based mechanosensing molecular modulators. Antigen specific synthetic notch receptors were tagged with a transcription factor (TF) intracellularly. Its activation can lead to gene expression upon antigen binding, *via* the subsequence release of TF upon cleavage (left). An apelin based SynNotch receptor tethered to a Cre or tTA recombinase intracellularly. Upon apelin-APJ interaction, the cleavage will release the recombinase and activate the designed gene of interest (right). **(B)** Schematic showing of ultrasound activation of engineered immune cells, Ultrasound can activate mechanosensitive ion channel expressing T cell and trigger gene activation through designed genetic circuits to specifically kill antigen expressing tumor cells (left). Macrophages were engineered with ultrasound-responsive components and the payload. Upon short-pulsed high intensity focused ultrasound (HIFU) sonication, the macrophages can be disrupted, which allows drug release into the tumor locus (right).

The development of the synthetic Notch (SynNotch) receptor is an example of integrating synthetic biology, mechanobiology, and immuno-engineering (Morsut et al., 2016) (Figure 4A). SynNotch receptors utilize the mechanical sensing property of Notch protein (Roybal et al., 2016a; Morsut et al., 2016). Mechanical forces can activate Notch receptors and cause subsequent proteolytic cleavage of their intramembrane domain. By fusing an antigen recognizing ScFv, a Notch receptor core domain, and an intracellular transcriptional factor, the SynNotch receptor can recognize a specific target antigen and become cleaved to release the transcriptional factor which can activate the designed CAR expression for a second antigen. These SynNotch-CAR T cells enable combinatorial antigen-sensing capabilities and higher specificity (Roybal et al., 2016b).

More recently, an apelin-based synthetic Notch receptor (AsNR) was developed to detect angiogenesis and treat solid tumors (Wang et al., 2020). Apelin is the endogenous ligand for a G-protein-coupled receptor (GPCR) apelin receptor (APJ), which is known to serve as a mechano-sensor that responds to stretching (Scimia et al., 2012). APJ is also an important regulator of blood pressure, angiogenesis, cardiovascular function, etc. By engineering immune cells with apelin, Notch core domain, and Cre recombinase or tetracycline transactivator (tTA) for cell activation, the engineered T cells can specifically target proliferating endothelial cells in tumors (Wang et al., 2020).

The mechanical tension generated at the IS can also be utilized in an anticancer drug delivery system. Lei and colleagues developed drug-loaded mesoporous silica microparticles equipped with force-sensitive DNA gatekeepers (Lei and Tang, 2020). The DNA, or mechano-sensor/gatekeeper, was linked to anti-CD3 and anti-CD28 antibodies so that once the particles interact with T cells, the tension generated from TCR activation will be transmitted to the sensor and, in turn, trigger the release of drugs.

### External mechanical modulator: Ultrasound

Ultrasound imaging, or sonography, has been used in medical practice for more than 50 years, helping physicians to evaluate and diagnose medical conditions (Kendall et al., 2007). Its simple and cost-effective procedure, along with the fact that it uses non-ionizing radiation, led ultrasound imaging to become the primary imaging modality in many cases including prenatal care. In recent years, ultrasound is gaining attention again as one of the promising external modulators for cell therapy because it can deliver mechanical energy into a confined volume of tissues anywhere in the body, in a remote and non-invasive manner. Moreover, safety guidance of ultrasound for clinical use is well-documented (Fowlkes, 2008), which could expedite the translation of the technique. While properly regulated ultrasound exposure would not pose any adverse effects on the human body, ultrasound is capable of inducing various thermal and mechanical bioeffects on cells and tissues when performed with adequate output power and duration (Miller et al., 2012). We briefly discussed immunotherapies based on ultrasound-generated thermal effects in the previous chapter, but in this manuscript, we will focus on mechanical aspect of ultrasound and its corresponding therapeutic applications. In general, pulsed ultrasound with relatively low intensity is used to induce mechanical bioeffects. For instance, numerous studies have demonstrated that low-intensity pulsed ultrasound (LIPUS) can accelerate bone fracture healing through mechanotransduction pathways (Della Rocca, 2009). Integrin-mediated FAK/MAPK/ERK signaling cascade was also found to be associated with LIPUS exposure (Whitney et al., 2012; Kusuyama et al., 2014; Sato et al., 2014), where it is often linked to upregulation of cyclooxygenase-2 (COX-2) and prostaglandin E2 (PGE2) production (Kokubu et al., 1999; Tang et al., 2006).

Another major cellular component that has been shown to respond to ultrasound is intracellular calcium concentration. Parvizi et al. (2002) reported that both transient and sustained intracellular calcium elevations can be observed in primary rat chondrocytes upon LIPUS exposure. Since then, ultrasound-induced calcium responses were monitored in different types of cells (Tyler et al., 2008; Hwang et al., 2014; Weitz et al., 2017; Yoon et al., 2020), and found to be directly linked with the activation of various mechanosensitive proteins (Ibsen et al., 2015; Yoon et al., 2017; Qiu et al., 2019; Yoo et al., 2022). Based on the observation that intracellular calcium dynamics can be regulated by ultrasound, a modular method has been developed to engineer immune cells with synthetic genetic circuits that can sense and transduce ultrasound stimulation. Hence, ultrasound can be applied remotely and non-invasively to these engineered cells to control their genetics and CAR protein expression to recognize specific antigens and kill the target tumor cells. Microbubble-mediated focused ultrasound system was used as a means of external modulator to activate mechanosensitive Piezo1 ion channels on HEK293T cells or primary T cells (Pan et al., 2018). The ultrasound-induced calcium influx through Piezo1 channel can activate calcium-sensitive pathways to drive the expression of designed target genes *via* synthetic genetic circuits (Figure 4B). While continuous evolution and optimization are required for *in vivo* and clinical applications, this study laid the foundation for developing remote and non-invasive modular systems for controllable cancer immunotherapy.

Recently, another study demonstrated the successful integration of ultrasound, as a mechanical energy-based external controller, for cell-based drug delivery systems. Xu et al. (2020) developed ultrasound-traceable and activatable cell bombs by encapsulating ultrasound reactive phase transformable perfluoropentane (PFP) with a chemotherapeutic agent, doxorubicin, into macrophages. The engineered macrophage can be imaged in real-time while actively homing to the tumor site. Once the therapeutic payload-containing cells accumulate at the tumor loci, the drug can be released in response to short-pulsed high intensity focused ultrasound (HIFU) sonification (Figure 4B).

### Challenges and future perspectives

There are still many challenges that lie ahead for the full fruition of this novel approaches. For instance, many known mechanotransduction pathways share their downstream cellular events with other signaling cascades. A good example of this case is intracellular calcium responses. The majority of mechanosensitive ion channels allow calcium influx upon mechanical perturbation; however, it cannot be distinguished from cytoplasmic calcium elevation *via* ligand-gated ion channels or receptors. Also, several mechanosensitive ion channels like TRPV4 can also be activated by non-mechanical inputs. Thus, calcium-dependent genetic transduction modules can suffer from high basal activations due to spontaneous calcium signaling which can accumulate over time. Immune cells are generally more active in calcium dynamics (Vig and Kinet, 2009), making them more vulnerable to non-specific activation unless well-controlled. In fact, the non-specific activation could also come from the overexpression of mechano-sensitive components in engineer cells since various mechanical cues are continuously present in the cellular microenvironments. Nevertheless, we believe that this is where the power of synthetic biology can contribute. In fact, controlling or suppressing non-specific activation has been one of major battle fields in synthetic circuit design. A variety of genetic logic circuits have been developed to provide high specificity and versatility (Xie et al., 2020). Similar approaches can be implemented to mechanosensitive synthetic circuits to address the background noise issue.

Next, further characterization and optimization for focused ultrasound will be needed specifically in the context of the mechanical energy-based modulation. Biophysical understanding of how acoustic waves interact with different cellular environment and mechanosensors will be a great interest which could potentially stimulate various innovative therapeutic applications. Systematic assessment for therapeutic dosage of ultrasound that corresponds to different mechanical bioeffects could also benefit the approach. Lastly, methods for real-time monitoring of ultrasound energy deposition and distribution can be further developed to offer better controllability and dosimetry.

## Conclusion

Historically, scientists have learned from nature and applied the principles to develop novel and innovative therapeutics. Although there has been a lot of progress in the field of mechanobiology in recent years, there is still more to explore, particularly related to immune system. In this aspect, immuno-engineering which is based on mechanobiology is the road less traveled with full of potential. Every year, there are newly identified mechano-sensitive proteins or mechanotransduction pathways reported replenishing the arsenal. One obvious advantage of this approach is that it can be readily combined with other cutting-edge synthetic biology strategies to provide an additional layer of specificity or controllability to the system. In addition, these approaches could be particularly powerful when they are incorporated with mechanical energy-based modalities such as focused ultrasound which offer unprecedented spatial and temporal precision to the therapeutic schemes, such as CAR T therapy. In fact, CAR T cells with precise control of its activation in space and time can alleviate the life-threatening on-target off-tumor toxicity.

In this review, we summarized the recent studies and progress in immune cell mechanotransduction, current designs for immuno-engineering, as well of applying mechanobiology principles to immuno-engineering. Lastly, we also discussed the challenges and future directions. Despite all the challenges, it is expected that each component of this mechanobiology-based immune-engineering, i.e., mechanical modulators, genetic/epigenetic regulating modules, and mechano-sensors, will continue to evolve for greater precision. We envision that a mechanobiology-based immuno-engineering strategy will lead to a paradigm shift in translational medicine and pave the way for future telemedicine approaches.
